# Flavor Preference Learning Increases Olfactory and Gustatory Convergence onto Single Neurons in the Basolateral Amygdala but Not in the Insular Cortex in Rats

**DOI:** 10.1371/journal.pone.0010097

**Published:** 2010-04-09

**Authors:** Bertrand Desgranges, Victor Ramirez-Amaya, Itzel Ricaño-Cornejo, Frédéric Lévy, Guillaume Ferreira

**Affiliations:** 1 Laboratoire de Comportement, Neurobiologie et Adaptation, INRA UMR 85, CNRS UMR 6175, Université Tours, Nouzilly, France; 2 Departamento de Neurobiología Conductual y Cognitiva, Instituto de Neurobiología, Universidad Nacional Autónoma de México, Querétaro, Querétaro, México; Max-Planck-Institut für Neurobiologie, Germany

## Abstract

The basolateral amygdala (BLA) and the insular cortex (IC) represent two major areas for odor-taste associations, i.e. flavor integration. This learning may require the development of convergent odor and taste neuronal activation allowing the memory representation of such association. Yet identification of neurons that respond to such coincident input and the effect of flavor experience on odor-taste convergence remain unclear. In the present study we used the compartmental analysis of temporal activity using fluorescence in situ hybridization for *Arc* (catFISH) to visualize odor-taste convergence onto single neurons in the BLA and in the IC to assess the number of cells that were co-activated by both stimuli after odor-taste association. We used a sucrose conditioned odor preference as a flavor experience in rats, in which 9 odor-sucrose pairings induce a reliable odor-taste association. The results show that flavor experience induced a four-fold increase in the percentage of cells activated by both taste and odor stimulations in the BLA, but not in the IC. Because conditioned odor preference did not modify the number of cells responding selectively to one stimulus, this greater odor-taste convergence into individual BLA neurons suggests the recruitment of a neuronal population that can be activated by both odor and taste only after the association. We conclude that the development of convergent activation in amygdala neurons after odor-taste associative learning may provide a cellular basis of flavor memory.

## Introduction

During food intake, smell and taste interact to generate the perception of flavor [1]. Olfactory and gustatory information are each subserved by different receptors and different neural systems but they converge in the amygdala and the ventrolateral frontal cortex in mammals [1], [2].More precisely, the basolateral amygdala (BLA) and the insular cortex (IC) represent two major areas in rodents for the integration of odor-taste associations as they receive both olfactory and gustatory afferents, in addition to visceral inputs [3], [4], [5]. However, cellular mechanisms of odor-taste interactions remain unclear. According to Hebb's proposal [6], associative learning could produce convergent neuronal activation in the regions involved in such an association, however this has not been demonstrated yet in single BLA or IC neurons after odor-taste association.

Lesion studies provide information about a differential role played by BLA and IC in the processing and/or memory of odor-taste association. Using conditioned odor preference (COP) as odor-taste association learning, recent studies indicate that amygdala lesions (including BLA) strongly impaired COP induced by repeated association of an odor with a sweet taste (saccharin, fructose or sucrose; [7]–[9]), whereas IC lesion did not affect this associative learning [8], [10]. Although odor and taste inputs converge in the BLA and the IC, the differential effect of BLA and IC lesions on COP suggested that COP learning may allow the development of convergent odor-taste neural activation in BLA neurons but not in IC neurons.

To test this hypothesis, we studied the effect of sucrose-COP experience on neuronal activation induced by odor and taste stimulations in the BLA and the IC, by using *the* compartmental *a*nalysis of temporal activity with *f*luorescence *i*n *s*itu *h*ybridization (catFISH) for Arc mRNA. With this method we can visualize, within a single brain, the neuronal populations activated by each or both stimulations, odor and taste [11], [12]. We found that 9 odor-taste pairings modify the hedonic value of the odor demonstrating a reliable odor-taste association. As hypothesized, the neuronal population activated by both odor and taste strongly increased in the BLA, but not in the IC, after this flavor experience. Interestingly, our results suggest that this greater odor-taste convergence in the BLA is based on the recruitment of a new population of previously silent neural units that acquired the ability to respond to both chemosensory inputs after repeated odor-taste association.

## Results

### Behavioral evidence of conditioned odor preference

**Figure 1:**
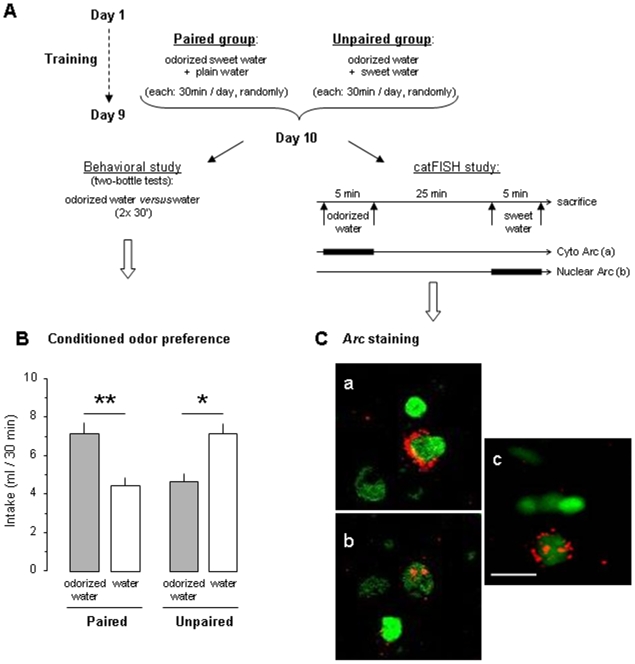
Conditioned odor preference and *Arc* expression in amygdala and insular cortex. (A) Schematic of the procedure used for behavioral and catFISH studies. (B) Consumption of odorized water (grey bar) or plain water (open bar) per 30 min during a two-bottle choice test. (C) Representative image from the basolateral amygdala showing Arc localization following stimulus presentation in a Paired animal. a) Neuron responding only to the first stimulation (odor) shows Arc staining (in red) in the cytoplasm surrounding the nucleus (counterstained green). b) Neuron responding only to the second stimulation (taste) shows dense Arc foci within the nucleus. c) Neuron responding to both odor and taste shows cytoplasmic and nuclear staining. Scale bar, 10 µm. **, *: intra-group difference (p<0.01; p<0.05).

We tested whether repeated paired presentations of an odor and sucrose induced COP for this odor (Fig. 1A). After 9 days of training, the Paired group preferred scented water over plain water (paired t-test, t_(9)_ = 4.0, p = 0.003) whereas the Unpaired group preferred plain over odorized water (t_(9)_ = 2.8, p = 0.02; Fig. 1B) like naïve animals (data not shown). These results indicate that 9 paired presentations of odor and sweet taste induce a reliable odor-taste association.

### Odor-taste association learning increased the coincident activation of individual neurons by odor and taste in the BLA, but not in the IC

After 9 days of training, the Paired and the Unpaired animals were exposed first to scented water for 5 min followed 25 min later to sucrose for 5 min and they were sacrificed immediately after (Fig. 1A). All the Paired and the Unpaired animals consumed the 6 ml of scented water and sucrose proposed. Given Arc expression dynamics [13]–[15], cells responsive to the olfactory (first) stimulation are the ones showing *Arc* staining restricted to the cytoplasm (Fig. 1C,a) whereas cells responsive to the gustatory (last) stimulation showed *Arc* staining restricted to the nucleus in the form of two intense nuclear foci (Fig. 1C,b). Cells responsive to both odor and taste stimuli present both cytoplasmic and nuclear *Arc* staining (Fig. 1C,c).

**Figure 2:**
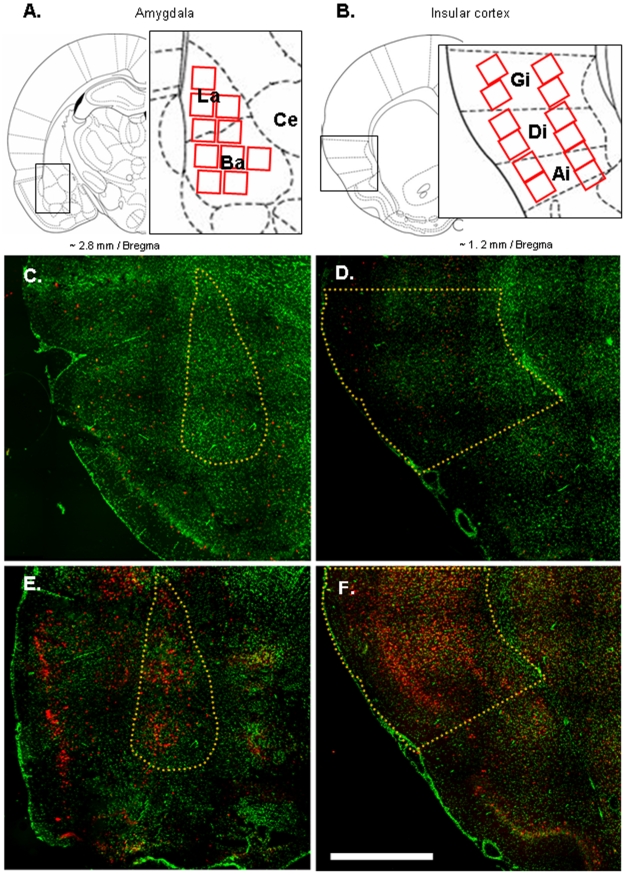
Examples of *Arc* induction in basolateral amygdala and insular cortex. (A, B) Schematic drawing of brain sections assayed at the level of (A) the basolateral amygdala, and (B) the insular cortex (adapted from Paxinos & Watson, 1998). Each red square represents the regions where image stacks were obtained. Ai: agranular zone of insular cortex; Ba: basal nucleus of amygdala; Ce: central nucleus of amygdala; Di: dysgranular zone of insular cortex; Gi: granular zone of insular cortex; La: lateral nucleus of amygdala. (C, D, E, F) Representative fluorescent images at the level of the basolateral amygdala (C, E) and insular cortex (D, F) using the Apotome microscope system (Zeiss). Green is the Sytox green nuclear staining and red is the Cy3 signal used to detect Arc mRNA. Images taken from the Caged control rats (C, D) and from the Paired animals exposed to odor and taste (E, F). The yellow lines delineate the basolateral amygdala and insular cortex. Scale bar, 1mm.

**Figure 3:**
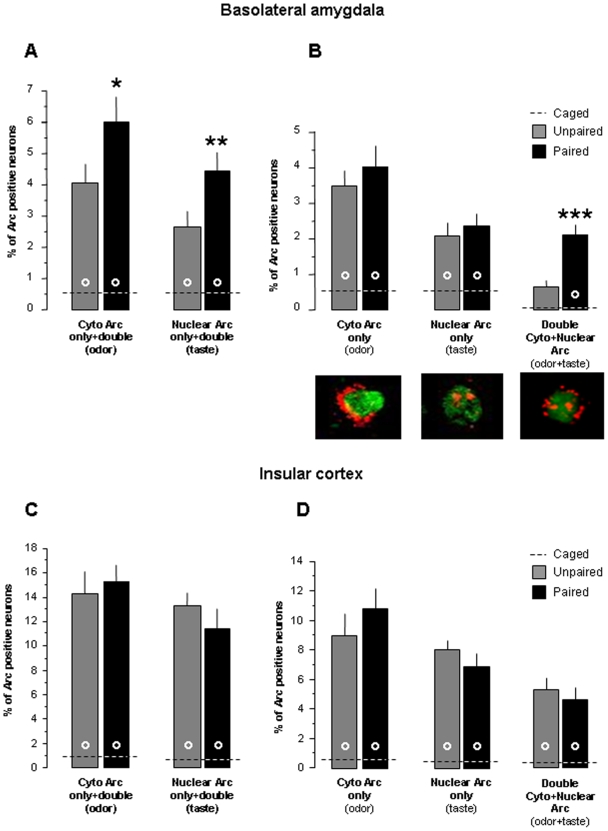
Flavor experience increase odor-taste convergence in basolateral amygdala but not in insular cortex. Percentage of Arc positive neurons in the basolateral amygdala (A,B) and in the insular cortex (C,D) after odor and taste presentations. Cytoplasmic responses correspond to the odor stimulation and nuclear responses correspond to the taste stimulation in the Paired and the Unpaired groups. Data are represented as means ± SEM. ***, **, *: different from the Unpaired group (p<0.001; p<0.01; p = 0.057). °: different from the Caged group (p<0.01).

In amygdala, *Arc* expression was predominantly found in the BLA (Fig. 2A,E). Odor and taste presentations induced a higher percentage of Arc positive neurons in BLA in the Paired and the Unpaired animals as compared to the Caged control animals that remained undisturbed in their home cage (F_(2,12)_>7, p<0.01 for all comparisons, except for the percent of double staining of the Unpaired group which did not differ from the Caged group; Fig. 2C,E and Fig. 3A,B). Moreover, the Paired group showed a higher percentage of BLA neurons with cytoplasmic (odor stimulation) or nuclear (taste stimulation) staining compared to the Unpaired group (p = 0.057 and p = 0.005, respectively; Fig. 3A). Interestingly, this effect represents a four-fold increase in the percentage of neurons with both cytoplasmic and nuclear staining in the Paired group as compared to the Unpaired group (p<0.0001; Fig. 3B). The percentage of neurons with either cytoplasmic staining only or nuclear staining only did not differ between the Paired and the Unpaired groups (p = 0.48 and p = 0.39, respectively; Fig. 3B). This revealed that more neurons were activated by both odor and taste in the animals that were previously exposed to the paired presentation of the stimuli.

*Arc* expression was found in all layers and subdivisions of the IC (Fig. 2B,F). As in the BLA, there was much less *Arc* activation in the IC of the Caged control animals than the stimulated animals, regardless from their previous experience (F_(2,12)_>9, p<0.01 for all comparisons; Fig. 2D,F and Fig. 3C,D). In contrast to what was observed in BLA, the pattern of Arc expression was not different between rats in the Paired and the Unpaired groups in all subcellular regions (p>0.2 for all comparisons; Fig. 3C,D).

## Discussion

By using *Arc* catFISH as a functional imager to visualize neuronal populations activated by two discrete sensory stimulations, we observed that the neuronal population responding to both odor and taste is increased in the BLA in animals previously experienced with COP training. More precisely, the underlying mechanisms in the BLA involved a four-fold increase of neuronal populations presenting coincident odor-taste activation.

The IC showed a higher percentage of Arc expression compared to BLA in both groups which could be related to somatosensory input of liquid consumption in addition to chemosensory input [16]. More importantly, a similar number of neurons responding to both odor and taste stimulations was found in the Paired and the Unpaired groups. This indicates that neuronal activation in the IC is independent of previous flavour experience. An alternative explanation would be that, in the Unpaired group, the IC is highly responsive to familiar condition. On the whole, IC would be non-specifically related to the associative processes, as previously suggested in an aversive paradigm [15]. It may be possible that convergent activation in the IC develops with additional learning, however, lesion studies had indicated that amygdala is crucial for COP whereas IC is not [7]–[10]. These results suggest that odor-taste convergence onto individual BLA neurons could be a cellular basis of flavor associative memory.

Our results also addressed a fundamental problem in behavioural neuroscience concerning the mechanisms underlying associative conditioning. According to Hebb's theory ([6], pp. 126–127), convergent activation of the associated stimuli may occur in the neural networks underlying associative learning. In other paradigms, fear conditioning and conditioned taste aversion, the conditioned stimulus (CS) and the unconditioned stimulus (US) have been shown to induce convergent activation in single BLA neurons during or immediately after learning [15], [17]–[19]. This convergent activation is suggested to result from strengthening the synapses, of either the CS [18] or the US [15], [19], involving the neural units that already respond to one of the stimuli used in the association. Here we found that COP did not change the neural ensembles responding to only one stimulus (either odor or taste) in BLA whereas it increased the neuronal population showing concurrent activation by odor and taste. This suggests that a population of previously silent neurons develops the ability to respond after the paired presentation of the stimuli. In this scenario, this new population may have received no (or weak) inputs from either odor or taste stimulus before COP training. When the odor or the taste is presented alone it fails to excite these BLA neurons (as evidenced by results of Unpaired group; Fig 3B). However, during COP training, when both stimuli were combined these neurons become excited by summation of stimulations, resulting in odor-taste convergence. After repeated odor-taste associations, each stimulus alone is sufficient to activate these neurons (Paired group; Fig. 3B). However, it is also possible that neurons that were previously responsive to only one stimulus (either odor or taste) became responsive to the other stimulus after association. In this case the system compensates for the reduction on the number of neurons responding to odor or taste and neurons that were previously silent became responsive to either of these sensory stimuli. In any case, one or more sensory inputs in previously silent cells become strengthened after COP [20].

Therefore, our results suggest a different mechanism of associative plasticity in comparison to the one reported in taste aversion and fear conditioning [15], [17]–[19]. It remains to be clearly established whether the difference is due to the nature of the learning experience (aversive versus appetitive) or the memory phase investigated (acquisition of one-trial learning versus retrieval of spaced training). Moreover, as COP requires several training trials to develop, further experiments will determine through the training sessions whether the development of convergent activation in BLA neurons is concomitant to the improvement of behavioural performance.

We cannot completely exclude the possibility that the higher *Arc* activation in BLA of the Paired animals was partly due to the unexpected situation of the test in comparison to training as previous reports indicate BLA activation by novel stimulation [21]. However, the behavioural results of the Paired group clearly suggest that this situation (presentation of the odor without the sweet taste) is not unexpected as a strong preference for the odorized water was obtained (Fig. 1B). In addition, in the present study, the percentage of cells expressing Arc induced by odor or taste stimulations was 3–6% of BLA cells. It was previously shown that a novel taste induced Arc expression in 10–12% of BLA cells and that several pre-exposure to the taste induced a 3-fold decrease of Arc expression [15] suggesting that the low percentage of BLA cells activated by our testing condition is related to the familiarity of the stimulus.

Moreover, the sparse Arc expression in the BLA could reflect the involvement of this structure in numerous behaviours, from processing positive reward based memories in appetitive situations to fear-based and aversive memories [22]. The sparse code for odor and taste in the BLA suggests that this structure may be important for memory storage, as suggested for other regions, such as the dentate gyrus in the hippocampus, were a sparse code has been observed using the catFISH method [23].

Our results suggests that the neuronal representation for odor in the BLA recruits a bigger neural ensemble than taste since a greater percentage of Arc expressing neurons is observed following odor stimulation as compare to taste stimulation in the BLA of the Unpaired animals (t = 3.7, p<0.02; see Fig. 3A). This effect may also be explained by the order of sensory presentation, however, similar Arc activation was previously reported for taste stimulations whatever the order of presentation [15]. The higher BLA activation induced by odor as compare to taste is consistent with behavioural studies showing that BLA is more critical for odor learning than for taste learning. Indeed, BLA lesion induced greater impairment in COP than in conditioned taste preference [9]. Similarly, pharmacological manipulations of the BLA have a more detrimental effect on conditioned odor aversion than conditioned taste aversion [24]–[26].

*Arc* protein is important for synaptic plasticity underlying memory formation. Disruption of Arc expression by antisense or *knock-out* technologies leads to a disruption of long term memory [27]–[29]. In the present study, *Arc* expression in BLA neurons with coincident odor-taste activation was boosted by previous learning of odor-taste association providing a cellular basis of flavor associative memory. The fact that we found Arc expression after several training trials suggests that the function of Arc is still required after the consolidation of the odor-taste association and that *Arc* could be important for the establishment of persistent forms of synaptic plasticity such as structural synaptic changes or the homeostatic preservation of the plastic change [30] required for the long term memory of odor-taste association.

## Materials and Methods

### Subjects

Male Wistar rats (∼60 days old, 270–320g; Janvier, France) were housed individually in polypropylene cages (34×29×17 cm) lined with abundant pine shavings and kept in a temperature (23°C) and light (7h00–19h00) controlled room. Food and water were provided *ad libitum* until the beginning of the behavioral procedure. Experiments were performed in accordance with French and European regulations concerning animal experimentation, including authorizations 006352 and A37801 from the French Ministry of Agriculture to perform experiments, and ECC directive 86/609/EEC.

### Behavioral procedure

Four days before the conditioning, rats were adapted to a water restriction schedule with two daily drinking sessions in their home cage from 10:00 to 10:30 am and from 4:00 to 4:30 pm. Then, the Paired group (n = 10) received 9 daily presentations of a solution containing the odorant and tasteless isoamyl acetate (iso 0.01%; banana scented solution; Sigma, France; [31]) mixed with the sweet taste sucrose (0.1M, 3.4%; Sigma, France) both diluted in water (Fig. 1A). The presentation of odor-taste mixture was distributed randomly between the morning and the afternoon drinking session and water was provided during the other daily session. The Unpaired group (n = 10) received 9 daily presentations of the banana-scented water (iso 0.01%) and the sweet water (0.1M sucrose). In this case also, the presentation of scented water and sweet water occurred randomly between the morning and the afternoon drinking session. The day after the end of training, COP was assessed in Paired and Unpaired groups during both the morning and the afternoon drinking sessions by providing a simultaneous two-bottle test between one bottle containing banana-scented water and another one containing plain water. The left/right position of the scented solution was reversed between the morning and the afternoon session.

As a reliable odor-taste association was obtained after 9 odor-sucrose pairings (see Fig. 1B), an independent group of animals were trained similarly for the catFISH study. But the day after the 9^th^ pairing session, Paired and Unpaired animals (n = 6 in each group) had access first to 6 ml of the banana-scented water (iso 0.01%) during 5 min, followed 25 min later by 5 min access to 6 ml of the sweet water solution (0.1M sucrose). All animals consumed both 6 ml available and thus received similar olfactory and gustatory stimulations. The taste sucrose was deliberately not presented before the banana odor in order to avoid that the reinforcing properties and the lasting post-ingestive consequences of sucrose interfere with the following odor stimulation. In order to establish the basal level of *Arc* expression during the catFISH experiment, an additional caged control group (Caged, n = 3) was used. It was constituted of animals that remained in their home cage undisturbed with food and water *ad libitum* until sacrifice. They were sacrificed at the same time as the Paired and the Unpaired groups.

### In situ hybridization and confocal analysis

Immediately after sucrose presentation on day 10, animals were killed, the brains were rapidly extracted, and flash frozen in isopentane equilibrated in ethanol-dry ice slurry in less than 180 s, ensuring that the killing procedure did not induce detectable *Arc* transcription. The brains were stored at −80°C before sectioning. Brains coronal hemisections containing the amygdala or the IC from 5 rats were obtained by using a stainless still rectangular matrix (Electron microscopy sciences ®) and molded in a block with Tissue-Tek OCT compound (Sakura Finetek, France), using a stainless still rectangular matrix (Electron microscopy sciences ®). Each block contained at least one brain from each group of rats (i.e. Paired, Unpaired and Caged groups), and the brain from each group was located in a different position in each block. A total of 3 blocks were produced. The blocks were sectioned into 20-µm sections using a cryostat (Leica, Paris, France) at −18°C, captured on slides and stored at −70°C. Regions containing complete BLA (−2.8 mm from Bregma, Fig. 2A; [32]) and IC (+1.2 mm from Bregma; Fig. 2B) were selected for in situ hybridization. Digoxigenin-labeled *Arc* riboprobes were generated from a modified cDNA plasmid (kindly provided by Dr P. Worley) and fluorescent in situ hybridization for *Arc* was carried out as described elsewhere [11], [14]. *Arc* signal was visualized using the cyanine 3 (CY3) TSA fluorescence system (Perkin-Elmer Life Sciences, Emeryville, CA) and the nuclei were counterstained with Sytox green (Molecular Probes, Eugene, OR). Images from stained slides were acquired using a Zeiss LSM 510 confocal microscope (Zeiss Mexico) with a 40×/1.3 NA oil immersion objective, using the 561 helium/neon laser to excite the CY3 signal, and the 488 argon laser to excite the Sytox green signal. Routinely the confocal parameters (i.e. offset and amplifier settings) are established in a cage control animal on the slide (which is the only position known to the experimenter) and those parameters are kept constant for imaging the rest of the brains in each slide. About 6–9 optical Z-sections ∼0.3 µm–thick were obtained from each BLA slide section (Fig. 2A) and 12 images per IC slide section (2 from either the superficial or the deep layer of each IC subdivisions, agranular, dysgranular and granular; Fig. 2B). Two-3 slides for each region of interest were selected per block. The superficial and deep layers of the IC were chosen first because double activation is more likely to occur in the deep (V and VI) and superficial (I, II and III) layers since their cells integrate information coming from different cortical and subcortical areas and project to different cortical areas while middle layers receive primarily input from the thalamus [33]. Secondly, in our condition, the superficial and deep layers of the IC showed the highest density of Arc expressing cells (although Fig. 2F shows activity in medial layers, the amount of Arc expressing cells is higher in the deep and superficial layers). Finally, a previous catFISH study showed differential information processing in deep and superficial layers of the IC [33].

Confocal image stacks were analyzed using the visilog® image analysis software, by one experimenter blind to the experimental conditions. The details of the classification analysis had been described before [11]–[14]. Briefly, using Sytox green nuclear counterstaining signal (appear green in the images; Fig. 1C and Fig. 2C–F) neuronal nuclei was identified while glial nuclei was discarded from the analysis. This nuclear classification has been previously validated using immunohistochemistry specific to neurons and glial cells (NeuN and GFAP staining, respectively; [34]). GFAP positive cells have been found to show a greater intensity of Sytox signal than NeuN positive cells. To minimize sampling errors and stereological concerns an optical dissector technique was used, in which only neuron-like cells found in the middle 20% of the stacks were included in the analysis [14]. By using this method, the minor variations in cell volume do not influence sampling frequency. Once neurons were identified, they were classified according to its cytoplasmic and nuclear Arc staining, detected with CY3 signal (Fig. 1C). Neuronal nuclei surrounded >60% with the CY3 signal in 4 or more Z-section plains were classified as Arc cytoplasmic positive. Neurons with two intense CY3 nuclear foci visible across 3 or more Z-section plains were classified as Arc nuclear positive cells. Finally, cells that fulfilled both criteria were classified as double activated cells (Fig. 1C).

For both BLA and IC, the percent of cells with *Arc* expression was not different between the different images (different layers and subdivisions of the IC) and the different sections. Therefore the data were pooled together for either the BLA or the IC. For each rat, the mean number of cells counted in the BLA for all groups was 643 (standard deviation of 93) and for the IC was 1001 (standard deviation of 179). There was no correlation between the number of counted cells and the percent of cells expressing *Arc* RNA for either the BLA or the IC (p>0.99 and p>0.75, respectively).

### Statistics

Data were analyzed using paired *t* tests and one-way ANOVA, followed by *post hoc* Fisher tests when appropriate. In all cases, *p*<0.05 was considered significant.
